# Immune response against ocular tissues after immunization with optic nerve antigens in a model of autoimmune glaucoma

**Published:** 2013-08-06

**Authors:** Stephanie C. Joachim, Sabrina Reinehr, Sandra Kuehn, Panagiotis Laspas, Oliver W. Gramlich, Mathias Kuehn, Iris Tischoff, Harald D. von Pein, H. Burkhard Dick, Franz H. Grus

**Affiliations:** 1 Experimental Eye Research Institute, Ruhr University Eye Hospital, Bochum, Germany; 2 Experimental Ophthalmology, Department of Ophthalmology, University Medical Center, Johannes Gutenberg University, Mainz, Germany; 3 Institute of Pathology, Ruhr University, Bochum, Germany; 4 Department of Neuropathology, University Medical Center, Johannes Gutenberg University, Mainz, Germany

## Abstract

**Purpose:**

In recent years, numerous studies have investigated the involvement of immunological mechanisms in glaucoma. Until now, it has not been determined whether the altered antibody pattern detected in patients is harmful to retinal ganglion cells (RGCs) or triggers disease formation in any way. In a model of experimental autoimmune glaucoma, RGC loss can be induced through immunization with certain ocular antigens. In the current study, the time course of the levels of autoreactivity against ocular tissues after immunization was examined.

**Methods:**

Intraocular pressure was measured regularly. Ten weeks after immunization with an optic nerve homogenate antigen (ONA), the number of RGCs was determined. *Immunoglobulin* G levels in aqueous humor were measured via enzyme-linked immunosorbent assay at the same time point. Serum from different time points was used to analyze the possible occurrence of autoreactive antibodies against the retina or optic nerve in this autoimmune glaucoma model. Additionally, optic nerve and brain sections were evaluated for possible pathological findings.

**Results:**

Intraocular pressure stayed within the normal range throughout this study. A continuous increase of autoreactive antibodies against the optic nerve and retina sections was observed. At 4, 6, and 10 weeks, antibody reactivity was significantly higher in ONA animals (p<0.01). Aqueous humor *immunoglobulin* G levels were also significantly higher in the ONA group (p=0.006). Ten weeks after immunization, significantly fewer RGCs were noted in the ONA group (p=0.00003). The optic nerves from ONA animals exhibited damaged axons. No pathological findings appeared in any brain sections.

**Conclusions:**

Our findings suggest that these modified antibodies play a substantial role in mechanisms leading to RGC death. The slow dissolution of RGCs observed in animals with autoimmune glaucoma is comparable to the slow progressive RGC loss in glaucoma patients, thus making this a useful model to develop neuroprotective therapies in the future.

## Introduction

For a long time, glaucoma-induced vision loss was considered to be the consequence of high intraocular pressure (IOP). Today, we know that the pathomechanisms underlying this disease are probably much more complex. Most likely, vascular dysregulation [1] or mitochondrial dysfunction [2] makes retinal ganglion cells (RGCs) more sensitive to stress [3] and possibly play a role in glaucoma disease mechanisms.

Almost 20 years ago, Wax and coworkers detected antibody alterations in sera of normal pressure glaucoma patients for the first time [4]. Since then, multiple studies have been able to confirm autoantibody patterns against retina and optic nerve antigens in patients with glaucoma [5-7]. A considerable immune response seems likely during glaucomatous disease progression. It has been suggested that certain autoantibodies bind to neuronal proteins and then inhibit the functional effects caused by their activity [8,9]. Tezel et al. showed that exogenous anti–heat shock protein (HSP) 27 antibodies can enter retinal cells, likely via receptor-mediated endocytosis, and trigger apoptotic cell death [8]. Possibly, the internalized HSP 27 antibodies cause a decreased ability of endogenous HSP 27 to stabilize actin cytoskeleton, thereby leading to cell apoptosis. Retinal dysfunction could be initiated by intravitreal injection of anti-gamma-enolase [10]. However, whether the changes in antibody reactivity are the cause or consequence of RGC loss is still unresolved.

Previous researchers investigating the effect of immunization with ocular antigens on RGCs observed ganglion cell and optic nerve fiber loss in these animals [11,12]. Recently, we immunized animals with optic nerve antigen homogenate (ONA) and observed a significant RGC loss 4 weeks later [13]. Elevated autoreactive antibodies against the retina, optic nerve, and brain were also noted at this time.

The aim of the study was to observe the long-term alterations in autoreactive antibody patterns. We found that the development of autoreactive antibodies continuously increases following ONA immunization. In addition, we measured significantly increased levels of *immunoglobulin* G (IgG) in the aqueous humor of these animals.

## Methods

The experiments were performed in conformity with the Association for Research in Vision and Ophthalmology statement for the Use of Animals in Ophthalmic and Vision Research; the study was approved by the animal care committee of Rhineland-Palatine (Koblenz, Germany). Adult male Lewis rats (Charles River, Sulzfeld, Germany) were randomly placed in one of the two study groups: One group was immunized with ONA, while the was injected with sodium chloride (control group, CON), as described below. Animals were housed in light- and temperature-controlled conditions and were provided with feed and water ad libitum. Detailed observations of possible neurological deficits and eye exams were performed regularly.

Animals were sacrificed by CO_2_ after 10 weeks. Eyes, including the optic nerve, were enucleated. Eyes were prepared as cross-sections and optic nerves were prepared as longitudinal sections. Brains were also harvested, fixed, and prepared for sectioning. Additionally, brains and spinal cords were obtained after 12 days from a subgroup of animals.

### Immunization of animals

Fresh bovine eyes were obtained from the local abattoir (Schlachthof Alzey, Alzey, Germany). For optic nerve antigen preparation, the optic nerves from 12 bovine eyes was dissected behind the optic nerve head, and the dura mater was removed. The untreated tissue was transferred to a cooled mortar and ground until it reached a pulverized texture. This powder was suspended in PBS. Rats were injected with 8 mg ONA in 500 µl Freund’s adjuvant and 3 µg pertussis toxin (both Sigma-Aldrich, Munich, Germany) [13]. Control group animals were immunized with equal volumes of Freund’s adjuvant and pertussis toxin in sodium chloride. Booster injections consisting of half the dose of the initial injection, were given after 4 and 8 weeks.

### Intraocular pressure measurements and funduscopies

IOP was measured in both eyes of 10 animals per group before and 2, 4, 6, and 10 weeks after immunization using a TonoPen® XL applanation tonometer (Medtronic, Baseweiler, Germany) [13,14]. Topical anesthesia was applied before performing the measurements (Novesine, OmniVision, Puchheim, Germany). Means were calculated from 10 single measurements per eye. On the same day, the retinas of the animals were examined through a surgical microscope (Zeiss, Jena, Germany) and pictures were taken for detailed evaluation [13,14]. Funduscopies were performed under general anesthesia with isoflurane/O_2_ and a topical anesthesia (Novesine, OmniVision).

### Retinal ganglion cell staining via cross-sections

To evaluate whether RGC loss occurred after immunization with ONA, the RGCs were specifically labeled with neuronal nuclei (NeuN) [15-17] or γ-synuclein [18-20]. Eyes were obtained 10 weeks after immunization from both groups (n=4 animals/group, 6 cross-sections/animal), fixed (4% paraformaldehyde), underwent sucrose treatment, and were embedded in Tissue Tek (Fisher Scientific, Schwerte, Germany). Retinal cross-sections were cut on a Cryostat (10 µm thick; Fisher Scientific) and mounted on Superfrost Plus slides (Fisher Scientific). Antigen retrieval was performed by heating tissue sections for 30 min in citrate buffer (pH 6.0; 95 °C). The sections were blocked with 10% donkey serum in 0.1% Triton-X100 in PBS (0.137 M sodium chloride, sodium phosphate, 1.76 mM potassium phosphate, 0.027 M potassium chloride, and water; pH 7.4; Santa Cruz, CA) for 10 min. Sections were then incubated at room temperature with a chicken polyclonal NeuN antibody (1:500, Millipore, Darmstadt, Germany) or rabbit polyclonal γ-synuclein antibody (1:100, Abcam, Cambridge, UK) overnight. Sections were visualized by donkey antichicken Cy3 (1:400, Millipore) or goat antirabbit Cy3 (1:400, Linaris, Germany) secondary antibody. All slides were mounted with antifade medium with fluorescent nuclear stain 4',6-diamidino-2-phenylindole (DAPI; Dianova, Hamburg, Germany). From each retinal section, two photos of the peripheral and central part of the retina were captured with an Axiocam HRc CCD camera on a Zeiss Imager M1 fluorescence microscope (Zeiss). The digitalized images were transferred to Corel PaintShop Photo Pro (V13, Corel Corporation, CA). Cutouts were made that included the ganglion cell layer (GCL). Subsequently, NeuN^+^ and γ-synuclein^+^ cells were counted using ImageJ software (V 1.43; NIH) in a masked fashion. Group comparison was performed after transferring the data to the Statistica Software program.

### Immunoreactivity against retina and optic nerve tissue

Blood drawn before and 2, 4, 6, and 10 weeks after immunization (n=6 samples/group and time points) was used to examine the possible antibody development against ocular tissues [13,21]. Eyes and optic nerves of healthy Lewis rats were fixed in 4% formaldehyde. Tissues were embedded in paraffin and cut into 1 µm thin cross-sections using a microtome (Reichert-Jung, Depew, NY). Sections were mounted on glass slides and treated with xylene. Tissue sections were rehydrated and pretreated with 0.3% hydrogen peroxide. Afterwards, incubation with target retrieval solution was carried out. Sections were incubated with 1% bovine serum albumin and 0.5% Triton-X100 to prevent nonspecific binding. Then the retinal cross-sections were incubated with serum (dilution of 1:200 in PBS at room temperature). The optic nerve sections were also incubated with serum samples from both groups (dilution of 1:750). Following this, slides were washed in PBS and incubated with secondary anti-IgG (H+L) antibody (dilution of 1:500). Sera were omitted for negative control staining. Color was developed by incubation with 3′,3′-diaminobenzidine and sections were counterstained with hematoxylin. The staining intensity was evaluated by two masked examiners from 0=no staining up to 3=severe staining [13,22]. Statistical analysis was performed using Satistica software.

### Measurement of immunoglobulin G levels in aqueous humor

Aqueous humor was collected 10 weeks after immunization and stored at −80 °C for later analysis. IgG levels were measured using a sandwich enzyme-linked immunosorbent assay (ELISA) kit (eBioscience, Frankfurt, Germany) according to the manufacturer’s instructions. In brief, samples of aqueous humor from both groups were prediluted 1,000-fold in assay buffer (n=6/group). Seventy-five microliters of assay buffer were inserted into each sample well. Twenty-five microliters of the prediluted sample were added to the appropriate wells. All samples were incubated overnight. The absorbance was read at 405 nm using a microplate reader (AESKU.Reader with Gen5 ELISA Software, AESKU.DIAGNOSTICS, Wendelsheim, Germany).

### Longitudinal optic nerve sections

Following enucleation, optic nerves were fixed in 4% formaldehyde and embedded in paraffin (n=6 nerves/group). Optic nerves were cut in longitudinal sections (5 µm) for Harris hematoxylin and eosin G (H&E), Nissl (Merck) [13], and luxol fast blue (LFB; Sigma Aldrich) staining using standard protocols to examine axonal pathology and possible infiltrates.

Additionally, a Bielschowsky silver stain was performed [23]. Briefly, the rehydrated sections were placed in 10% silver nitrate solution at 40 °C for 15 min. After adding ammonium hydroxide to the silver nitrate solution, the slides were immersed in it and stained in an incubator at 40 °C for 30 min. Slides were then placed in developer working solutions for about 1 min. To stop the reaction, the slides were dipped in 1% ammonium hydroxide for 1 min. Then, the slides were placed in 5% sodium thiosulfate for 5 min. Finally, slides were dehydrated using 95% ethyl alcohol, absolute alcohol, and xylene, and then covered with mounting medium.

### Brain sections

Brains and spinal cords were obtained 2 (n=5/group) and 10 weeks after immunization (n=9/group). The brains and spinal cords were immersion-fixed in 4% buffered formalin; they were paraffin embedded, and then 4 µm thick sections were cut for H&E and LFB/periodic acid–Schiff (PAS) stain, as well as for immunohistochemistry. H&E and LFB/PAS stain were performed using standard protocols, as described previously [13].

Using the Dako autostaining device (Dako Autostainer Plus, Dako, Hamburg, Germany), horizontal sections of the brains and transversal sections of the spinal cords were immunostained with monoclonal antibodies to CD68 (clone PG-M1, dilution 1:600; Dako), which is a marker for macrophages, and CD45 (clone 2B11+PD7/26, dilution 1:500, Dako), which is a marker for lymphocytes. Processing by the Autostainer program comprised 5 min in peroxidase block (Dako), 30 min incubation in primary antibody, 30 min incubation in Histofine secondary reagent (Dako), and 10 min incubation in 3′,3′-diaminobenzidine. After completion of the staining procedure, the stained slides were removed from the device and counterstained for 5 min in Hemalaun.

### Statistics

Data are presented as mean±standard error of the mean (SEM), unless otherwise noted. The two groups, ONA and CON, were compared via a two-tailed Student *t* test using Statistica software (V10.0; Statsoft; Tulsa, OK). Null hypotheses were rejected at p<0.05.

## Results

### Course of intraocular pressure

**Figure 1:**
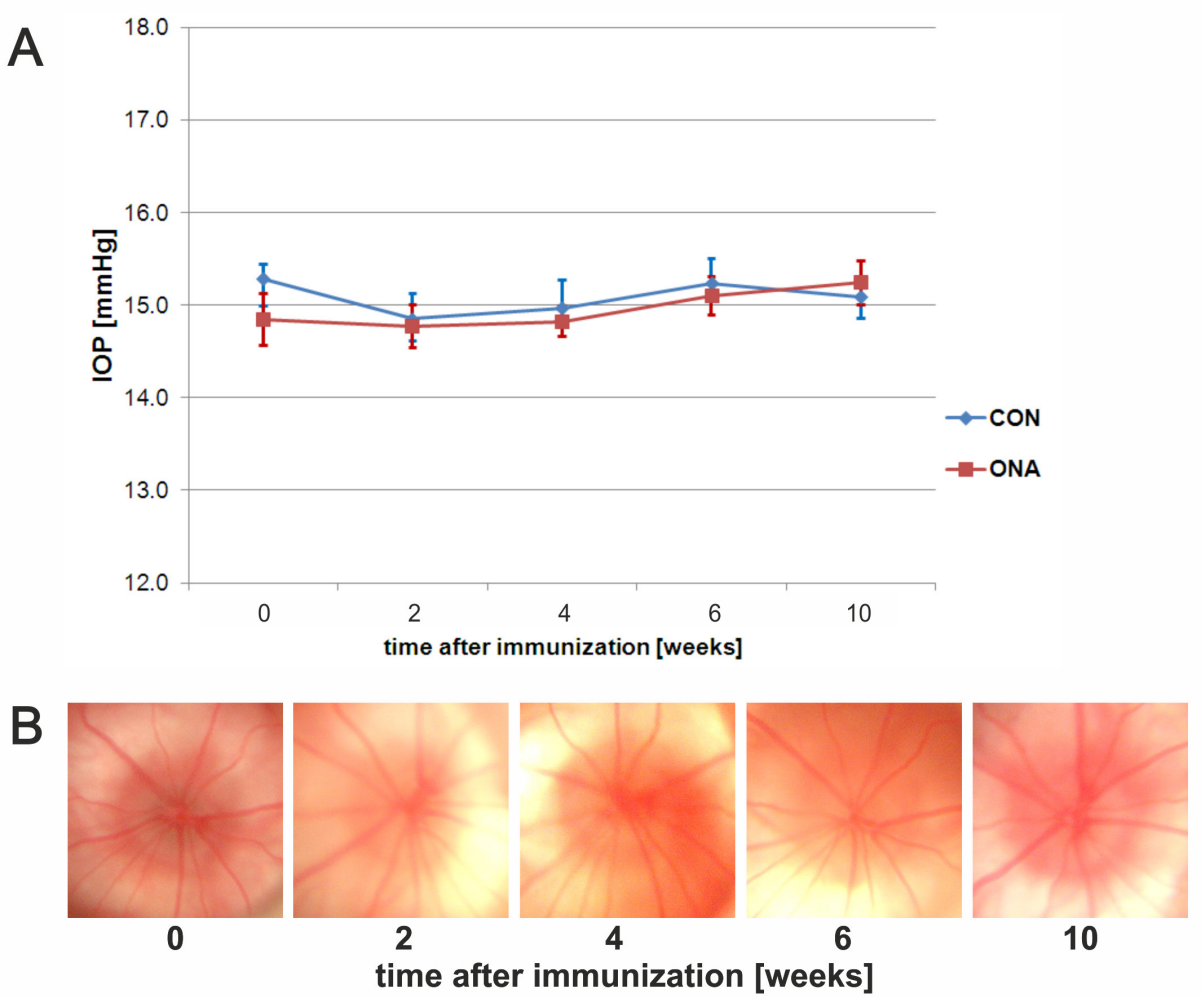
Intraocular pressure and fundus photos. **A**: Intraocular pressure (IOP) in controls (CON) and animals immunized with optic nerve homogenate (ONA). IOP was measured before (=0) as well as 2, 4, 6, and 10 weeks after immunization (n=10 animals/group). No significant difference between groups was observed (p>0.9 at all points in time). **B**: Exemplary fundus photos of an ONA animal before and 2, 4, 6, and 9 weeks after immunization. No abnormalities could be detected during the study. Values represent mean±standard error of the mean (SEM).

Both groups featured a mean IOP around 15 mmHg at the beginning of the study (mean±SEM: CON=15.3±0.2; ONA=14.8±0.3). The ocular pressure stayed within this range throughout the study. No significant differences could be found in IOP between groups over the duration of the study (p=0.9; Figure 1A). We could not detect any abnormal signs, such as bleeding or vessel obliteration, on fundus pictures from ONA and CON animals during the study (Figure 1B).

### Retinal ganglion cell loss after optic nerve homogenate antigen immunization

**Figure 2:**
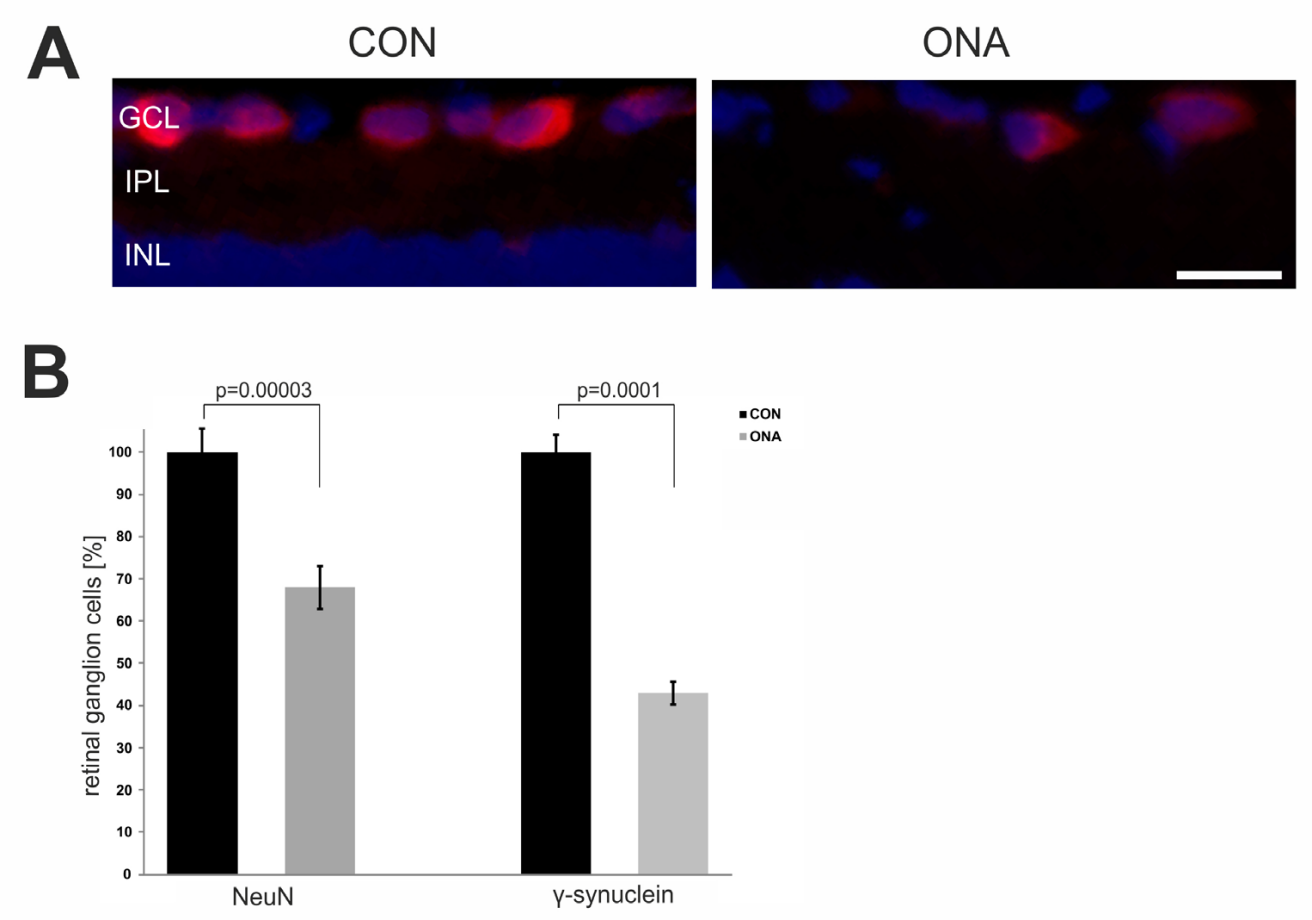
Evaluation of retinal ganglion cell loss in animals immunized with optic nerve homogenate. **A**: Exemplary photos of retina cross-sections of the control (CON) and the optic nerve homogenate antigen (ONA) group stained with the ganglion cell marker NeuN (red) and 4',6-diamidino-2-phenylindole (blue). **B**: Percentage of loss of NeuN^+^ and γ-synuclein^+^ cells in the ONA group compared to controls (n=4 animals/group). Significantly fewer retinal ganglion cells (RGCs) could be noted in the ONA group compared to CON (NeuN: p=0.00003; γ-synuclein: p=0.0001). The Student *t* test was performed to calculate the p values. Values represent the mean±standard error of the mean (SEM). Abbreviations: GCL=ganglion cell layer, IPL=inner plexiform layer, INL=inner nuclear layer (scale bar: 20 µm).

Ten weeks after immunization, RGC loss was determined by immunostaining with NeuN and γ-synuclein, two RGC-specific markers. We noted a 32% loss of NeuN^+^ cells on retinal cross-sections (p=0.00003). Regarding γ-synuclein^+^ cells, this loss was even greater. More than 50% of the γ-synuclein^+^ cells in the ONA group had died (p=0.0001). In conclusion, the ONA immunized animals had significantly fewer RGCs at 10 weeks (Figure 2B).

### Development of autoantibodies against ocular tissue

**Figure 3:**
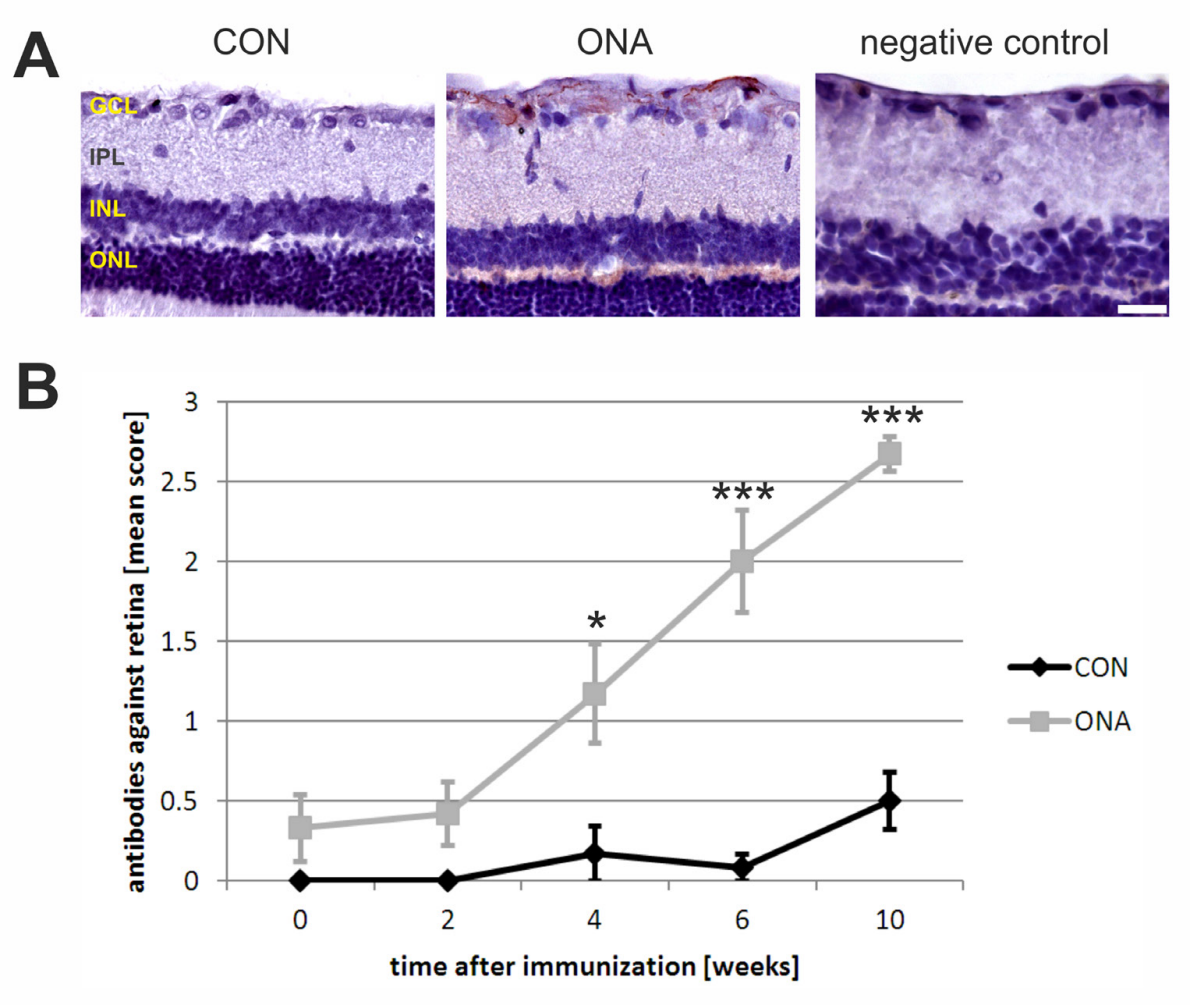
Increase in autoreactive antibody levels against the retina. **A**: Exemplary retinal cross-sections incubated with serum from a control (CON; left) and an optic nerve homogenate antigen (ONA) animal (middle) at 10 weeks (n=6/group). While basically no staining was observed on CON slides, strong staining due to autoantibody binding from ONA sera occurred. These antibodies bound especially to the nerve fiber and the ganglion cell layer (GCL). The negative control (right) was incubated only with secondary antibody and showed no staining. **B**: The diagram shows the mean autoantibody levels against the retina at different points in time. A continuous increase in autoantibodies was noted in the ONA group, while levels in the CON group stayed around baseline. At 4 (p=0.02), 6 (p=0.0002), and 10 weeks (p=0.000001), the mean antibody score in the ONA group was significantly higher than in the CON group. The Student *t* test was performed for every time point. Values represent mean±standard errors of the mean (SEM). Abbreviations: IPL=inner plexiform layer, INL=inner nuclear layer, ONL=outer nuclear layer (*: p<0.05; ***: p<0.001; scale bar: 50 µm).

The development of autoreactive antibodies against the retina and optic nerve was analyzed before as well as 2, 4, 6, and 10 weeks after the initial immunization. Regarding retinal cross-sections a continuous increase in autoreactive antibodies was observed up to 10 weeks after immunization (Table 1; Figure 3B). Before immunization (0 weeks), practically no staining was noted on retinas incubated with serum from the ONA (mean score: 0.33±0.21) or CON group (mean score: 0±0; p=0.1). Two weeks later, the mean scores of both groups were still comparable (ONA: 0.42±0.20; CON: 0±0; p=0.1). Four weeks after immunization, significant differences could be observed between the ONA (1.17±0.31) and CON groups (0.17±0.17; p=0.02). At 6 weeks, the ONA group showed a further increase in staining intensity (2.00±0.32), while the CON group still had low antibody levels (0.08±0.08; p=0.0002). A significant difference in mean scores was also noted at 10 weeks (ONA: 2.67±0.12; CON: 0.50±0.18; p=0.000001). Autoreactive antibodies in ONA sera especially bound in the nerve fiber and the ganglion cell layer (Figure 3A).

**Table 1:** Levels of autoreactive antibodies against retina and optic nerve.

**A: weeks**	**CON**	**ONA**	**p-value**
**0**	0±0	0.33±0.21	0.1
**2**	0±0	0.42±0.20	0.1
**4**	0.17±0.17	1.17±0.31	**0.02**
**6**	0.08±0.08	2.00±0.32	**0.0002**
**10**	0.50±0.18	2.67±0.11	**0.000001**

**B: weeks**	**CON**	**ONA**	**p-value**
**0**	0.08±0.08	0.08±0.09	0.9
**2**	0.17±0.17	0±0	0.3
**4**	0.25±0.17	1.50±0.22	**0.02**
**6**	0.50±0.26	2.17±0.17	**0.0003**
**10**	0.33±0.17	3.00±0	**0.00001**

**A**: Mean scores for autoreactive antibodies against retinal tissue. **B**: Mean scores for autoreactive antibodies against optic nerve tissue. Mean scores for autoreactive antibodies against ocular tissues. Serum samples obtained before (0 weeks) and two, four, six, and ten weeks after immunization from ONA and control (CON) animals were used for tissue incubation (n=6 samples/group). P values were calculated using Student *t* test. Values are mean±SEM. Significant p values are in bold.

**Figure 4:**
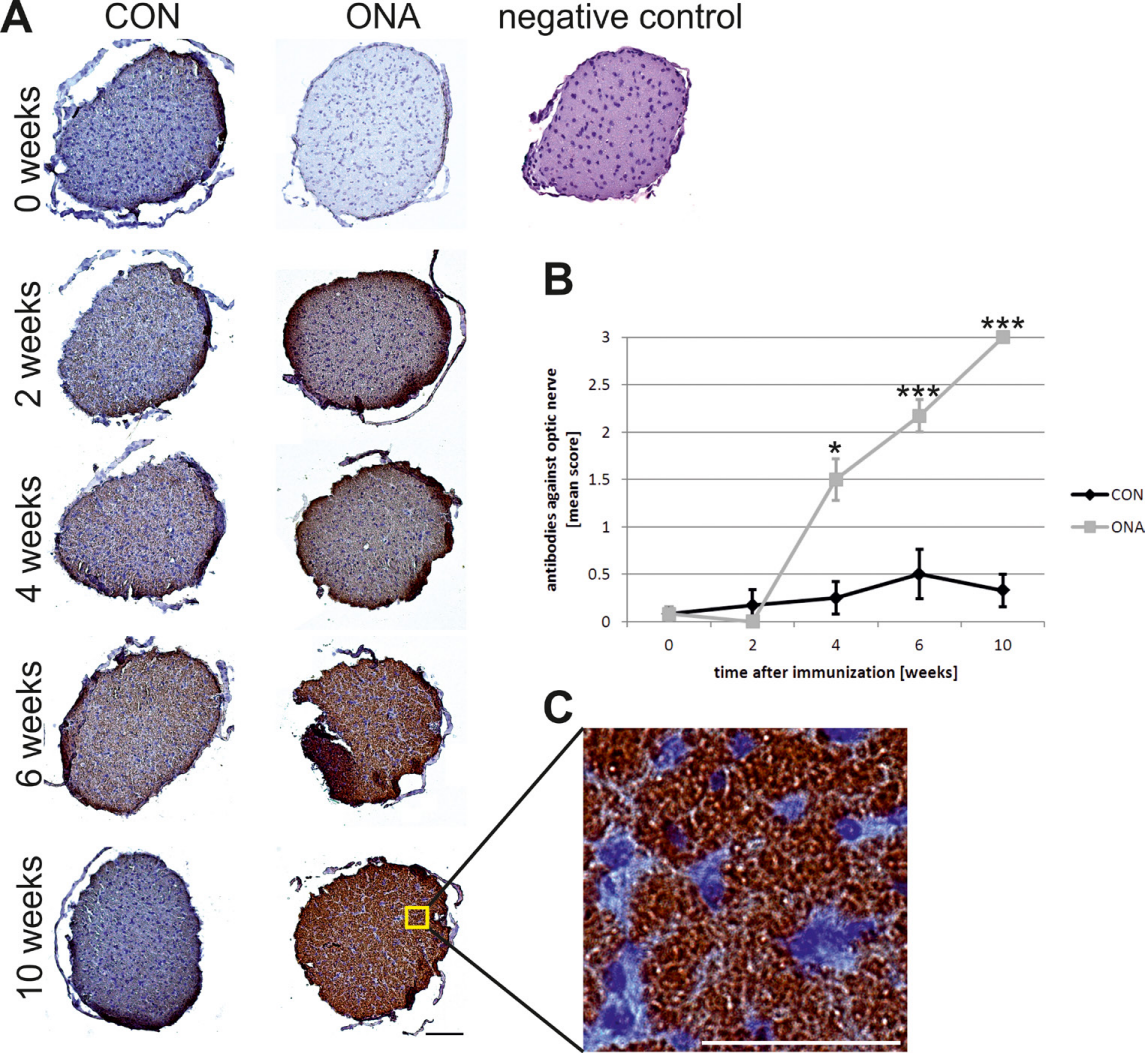
Development of autoreactive antibodies against the optic nerve. **A**: Optic nerve sections were incubated with CON or optic nerve homogenate antigen (ONA) serum (n=6/group). No staining was observed on optic nerve sections before immunization. The staining intensity on sections incubated with ONA serum continuously increased up to 10 weeks, while few staining signals were observed for CON serum at 4 and 6 weeks and not at the other points in time. At 4 (p=0.02), 6 (p=0.0003), and 10 weeks (p=0.00001), the ONA group had a significantly higher antibody score compared to controls. A negative control (right) was incubated only with secondary antibody and did not show any positive staining. **B**: Mean autoantibody levels against optic nerve before and up to 10 weeks after immunization. Both groups were compared at every point in time using the Student *t* test. **C**: Higher magnification of nerve section incubated with ONA serum obtained at 10 weeks. Distinct antibody binding was observed. Values represent mean±standard error of the SEM; *: p<0.05; ***: p<0.001; scale bar in A 100 µm and in C 50 µm).

An increasing binding reactivity against optic nerve sections was also noted for the ONA group during the course of the study (Table 1; Figure 4B). Before (ONA: 0.08±0.09; CON: 0.08±0.08; p=0.9) and 2 weeks after immunization (ONA: 0±0; CON: 0.17±0.17; p=0.3), no difference in staining intensity was noted between the two groups. At 4 weeks, significantly higher antibody scores were observed in the ONA group (1.50±0.22) compared to controls (0.25±0.17; p=0.02). Six weeks after immunization, differences in antibody scores had further increased (ONA: 2.17±0.17; CON: 0.50±0.26) and were statistically significant (p=0.0003). At 10 weeks, another gain in autoreactive antibodies was noted in the ONA group (ONA: 2.67±0.12; CON: 0.50±0.17; p=0.00001; Figure 4A,B).

### Increased immunoglobulin G levels in aqueous humor

**Figure 5:**
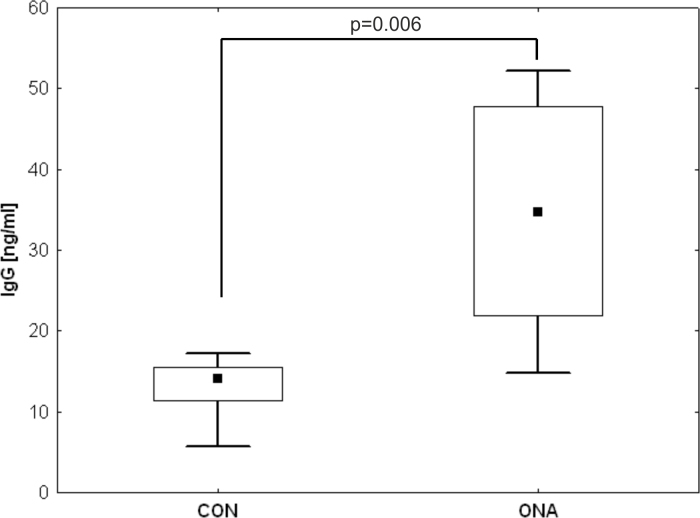
Increased immunoglobulin G levels in the aqueous humor of optic nerve homogenate antigen animals. The aqueous humor samples from control (CON) and optic nerve homogenate antigen (ONA) animals were tested for immunoglobulin (IgG) levels 10 weeks after immunization via enzyme-linked immunosorbent assay (ELISA; n=6/group). In the samples of ONA animals, a significantly higher amount of IgG could be detected compared to CON samples (ONA: 34.3±14.4 ng/ml; CON: 13.0±4.1 ng/ml; p=0.006).

IgG antibody levels were measured in aqueous humor 10 weeks after immunization via ELISA. A mean IgG level of 34.3±14.4 ng/ml was measured in the ONA group, while the mean IgG level in aqueous humor of the CON group was only 13.0±4.1 ng/ml. We observed significantly higher IgG levels in the ONA group (p=0.006; Figure 5).

### Damaged axons in optic nerve sections

**Figure 6:**
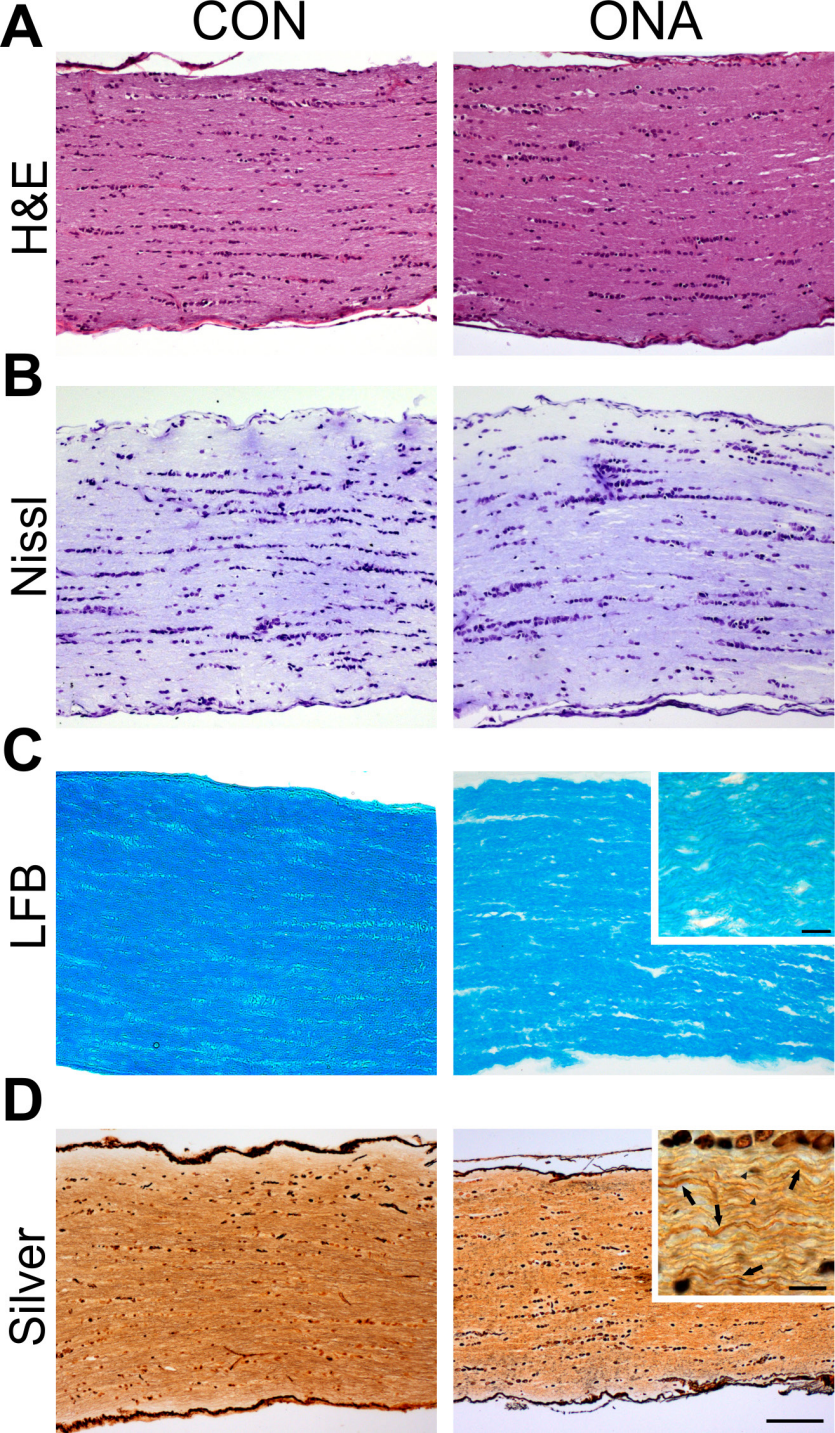
Optic nerve pathology in optic nerve homogenate antigen animals. **A**: Longitudinal optic nerve sections from both groups (control [CON] and optic nerve homogenate antigen [ONA]) stained with hematoxylin and eosin (H&E) showed no inflammatory cells (n=8/group). **B**: Optic nerve sections were also stained with Nissl. In neither group were cellular abnormalities noted, but the axons of ONA nerves were more disorganized (n=8/group). **C**: Luxol fast blue (LFB) staining was applied to assess signs of demyelination (n=8/group). Particularly in the magnified image, it can be clearly noted that axons of the ONA group are more disorganized, but still appear to be myelinated. **D**: Nerves were stained with Bielschowsky silver impregnation for axon evaluation (n=8/group). In ONA sections, many more damaged or swollen axons were noted. In the magnified section, arrows indicate swollen axons and arrowheads injured axons (scale bar 100 µm and higher magnification 10 µm).

H&E-stained optic nerve sections from both groups showed no infiltrates 10 weeks after immunization (Figure 6A). Nerve sections were stained with cresyl violet to evaluate the general structure of the nerves (Figure 6B). ONA nerves were much more disorganized. LFB-stained ONA nerves showed a disorganization of axons (Figure 6C). They appeared to be less regularly structured than the CON nerves, but were not completely demyelinated. Additionally, Bielschowsky’s silver impregnation was performed to evaluate axonal damage after immunization (Figure 6D). Many axons of ONA nerves appear to be swollen (arrows) or injured (arrowheads). In summary, the axonal structure of ONA animals was not as well preserved and several axons were damaged, but no cell infiltrates or massive infiltration was noted.

### Brain sections without pathological findings

**Figure 7:**
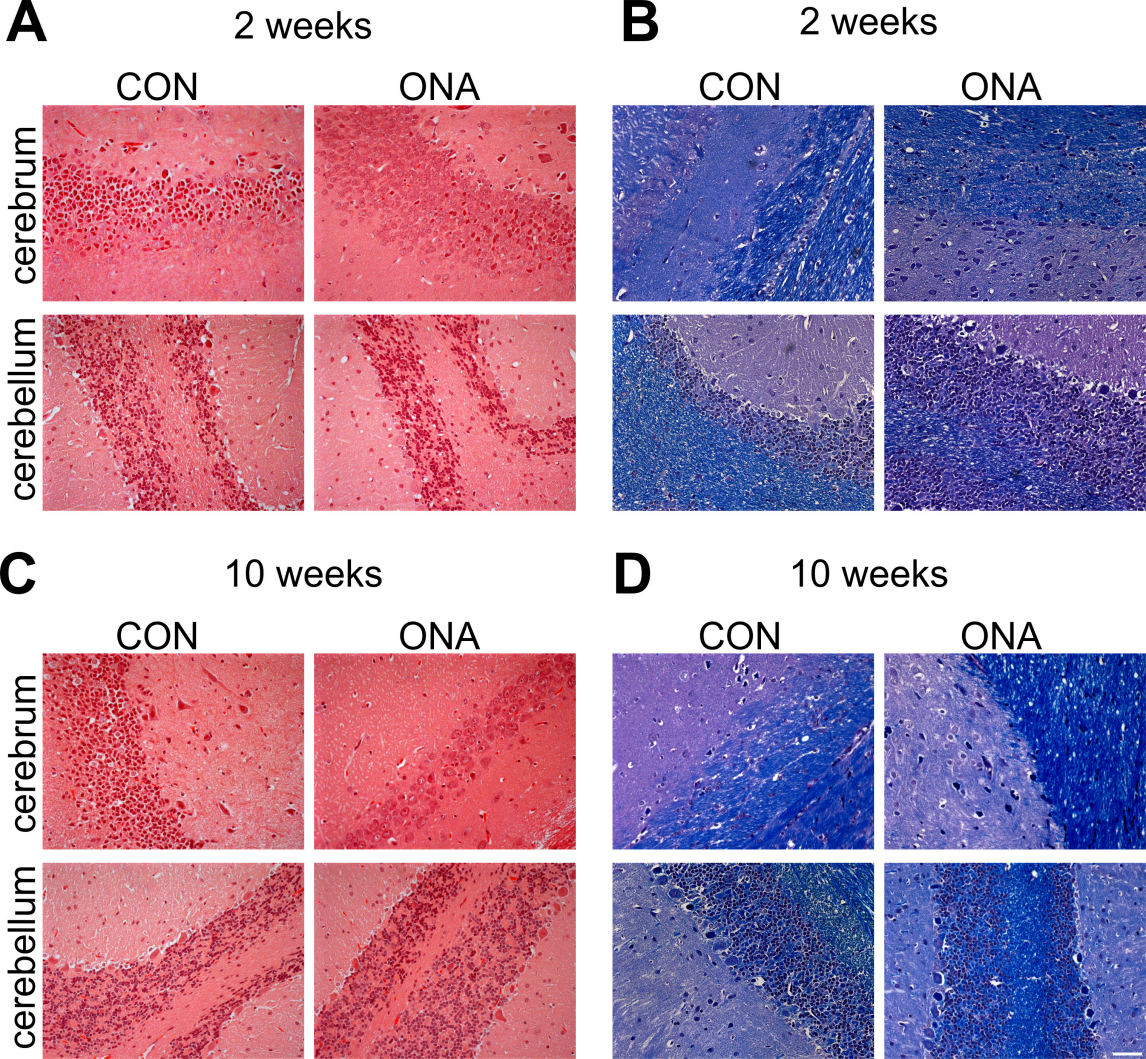
Unaffected brain sections. **A**: Exemplary hematoxylin and eosin (H&E) stained brain sections of control (CON) and optic nerve homogenate antigen (ONA) animals 2 weeks after immunization (n=5/group). No cellular infiltrates were noted in sections from either group. **B**: luxol fast blue/periodic acid–Schiff (LFB/PAS) stained sections were obtained at 2 weeks. Normal myelin was observed in both groups. **C**: Representative images were taken from horizontal brain sections of CON and ONA animals 10 weeks after immunization and stained with H&E (n=9/group). No infiltrates were noted at this later time point. **D**: LFB/PAS-stained brain sections were obtained at 10 weeks. At this time point, no demyelination was observed and no demyelinating plaques were noted. In summary, brain sections appeared to be without pathological findings at both time points (scale bar: 50 µm).

Sections of the central nervous system (CNS) were evaluated after 2 and 10 weeks to exclude any pathological changes in this region. Therefore, sections were stained with H&E, LFB/PAS, and markers for macrophages (CD68) and lymphocytes (CD45). Twelve days after immunization, neither demyelinated lesions nor mononuclear cell infiltrates consisting of macrophages and/or lymphocytes were detectable in the rat brains by routine histology (Figure 7A,B) or immunohistochemistry with monoclonal antibodies to CD68 and CD45 (data not shown). Ten weeks after immunization, the analyzed CNS sections still appeared to lack abnormalities (Figure 7C,D). This is in accordance with the previous findings of our group, where we analyzed brain sections 4 weeks after ONA immunization and did not observe any pathological findings [13].

## Discussion

In this model of experimental autoimmune glaucoma, loss of RGCs in the retina and damage to optic nerve fibers were induced through immunization with ONA (Figure 2 and Figure 6). Additionally, the development of autoreactive antibodies against ocular tissues was evaluated up to 10 weeks after immunization (Figure 3 and Figure 4). Interestingly, the level of autoreactive antibodies against ocular tissues continuously increased from 4 up to 10 weeks. Increased IgG levels could also be observed in aqueous humor.

In a previous study by our group, we were able to show that animals develop antibodies against retina and optic nerve up to 4 weeks after ONA immunization [13]. ONA immunization likely leads to a complex immune response, for example, activation of macrophages and lymphocytes and tissue stress. In the current study, we wanted to investigate the long-term effects of ONA immunization on autoantibody occurrence. We observed a continuous increase in autoreactive antibody levels up to 10 weeks after the initial immunization and significantly higher IgG levels in aqueous humor of these animals. Additionally, more RGCs died in the current study than in the previous one. Interestingly, however, the most severe cell death seemed to take place within the first four weeks after the initial immunization. At 10 weeks, about 32% of NeuN^+^ RGCs had died, while a loss of about 25% was already noted at four weeks. It appears that the initial immunization is more critical for the RGCs than the booster immunizations at 4 and 8 weeks.

In a mouse model for Batten disease, a neurodegenerative disorder, IgGs did not appear to be produced inside the CNS, but rather seem to be part of a systemic immune response. Autoantibodies were noted in the cerebrospinal of Cln3^−/−^ mice, as well as IgG depositions within the CNS [24]. These circulating IgGs can probably gain access to the CNS. This systemic production of IgGs also seems to apply to our model, since autoreactive antibodies significantly increased following immunization. These antibodies could enter the eye via corrupt or permeable regions of the blood-retina barrier (BRB). Although the retina is considered to be immunologically privileged, there seem to be several mechanisms that lead to a breakdown of the BRB, such as abnormal glia activity [25]. In our study, we noted higher levels of IgG in the aqueous humor of immunized animals (Figure 5), which might also be the result of a compromised BRB.

Patients with Lewy body–associated dementia, a neurodegenerative disease, have high levels of autoantibodies against, for example, myelin oligodendrocyte glycoprotein (MOG) and myelin basic protein (MBP). It has been postulated that these specific autoantibodies or B-cells can enter the CNS [26] due to a damaged blood-brain barrier [27]. We consider the possibility that systemic produced autoantibodies can pass through the partially damaged BRB in this model of autoimmune glaucoma. These autoantibodies then directly cause RGC death, possibly through antibody-dependent cytotoxicity [28] or trigger processes leading to cell loss. It has been reported that autoantibodies are able to penetrate living cells and initiate apoptotic processes [29].

Our study indicates that autoantibodies could trigger processes that lead to ganglion cell loss, such as complement activation. Some of these processes can also be observed in multiple sclerosis, where many demyelinating lesions are characterized by antibody depositions and complement activation [30]. Antibody depositions and consequent complement activation could also play a role in neuropathies. Complement-mediated nodal disruption might be involved in the development of certain neuropathies associated with autoantibodies against gangliosides [31]. AMPA receptor (GluR 3) antibodies haven been shown to bind to neuronal cells [32]. Similar disease-relevant autoimmune processes could lead to RGC death in our animal model. Therefore, the role of the complement system should be further investigated in the autoimmune glaucoma model, especially since previous studies showed a possible participation of the complement system both in rats [33] and in human glaucoma eyes [34].

In summary, the findings of this study support the claim that antibodies can play a crucial role in events leading to the apoptosis of RGCs. Increased levels of autoreactive antibodies against the retina and optic nerve were observed after immunization, as well as increased IgG levels in aqueous humor. We assume that the immune system plays a significant role in this model of experimental glaucoma.
